# Evaluation of suspected malignant hyperthermia events during anesthesia

**DOI:** 10.1186/1471-2253-13-24

**Published:** 2013-09-23

**Authors:** Frank Schuster, Stephan Johannsen, Daniel Schneiderbanger, Norbert Roewer

**Affiliations:** 1 Department of Anesthesia and Critical Care, University of Wuerzburg, Oberduerrbacher Str. 6, D-97080 Wuerzburg, Germany

**Keywords:** Malignant hyperthermia, In vitro contracture test, Succinylcholine, Volatile anesthetics

## Abstract

**Background:**

Malignant hyperthermia (MH), a metabolic myopathy triggered by volatile anesthetics and depolarizing muscle relaxants, is a potentially lethal complication of general anesthesia in susceptible patients. The implementation of modern inhalation anesthetics that research indicates as less potent trigger substances and the recommended limitations of succinylcholine use, suggests there may be considerable decline of fulminant MH cases. In the presented study, the authors analyzed suspected MH episodes during general anesthesia of patients that were referred to the Wuerzburg MH unit between 2007 and 2011, assuming that MH is still a relevant anesthetic problem in our days.

**Methods:**

With approval of the local ethics committee data of patients that underwent muscle biopsy and in vitro contracture test (IVCT) between 2007 and 2011 were analyzed. Only patients with a history of suspected MH crisis were included in the study. The incidents were evaluated retrospectively using anesthetic documentation and medical records.

**Results:**

Between 2007 and 2011 a total of 124 patients were tested. 19 of them were referred because of suspected MH events; 7 patients were diagnosed MH-susceptible, 4 MH-equivocal and 8 MH-non-susceptible by IVCT. In a majority of cases masseter spasm after succinylcholine had been the primary symptom. Cardiac arrhythmias and hypercapnia frequently occurred early in the course of events. Interestingly, dantrolene treatment was initiated in a few cases only.

**Conclusions:**

MH is still an important anesthetic complication. Every anesthetist must be aware of this life-threatening syndrome at any time. The rapid onset of adequate therapy is crucial to avoid major harm and possibly lethal outcome. Dantrolene must be readily available wherever MH triggering agents are used for anesthesia.

## Background

Malignant hyperthermia (MH) is mostly an inherited subclinical myopathy triggered by volatile anesthetics and depolarizing muscle relaxants in susceptible individuals, leading to a potentially lethal hypermetabolic reaction of skeletal muscle due to a disturbance of myoplasmic calcium homeostasis. Characteristic clinical signs of MH during a general anesthesia include hypoxemia, hypercapnia, tachycardia, muscular rigidity, acidosis, hyperkalemia and hyperthermia [1]. While expected genetic predisposition for MH is stated to be as frequent as 1:2.000, the prevalence of MH episodes varies regionally from 1:10.000 to 1:220.000 [2,3]. In contrast to fulminant MH episodes, abortive courses might occur more frequently, but are difficult to diagnose due to the alleviated symptoms.

Recent developments in anesthesiology apparently have lead to a decrease in severe MH crisis over the last years: Halothane, a potent MH triggering agent, is no longer used in clinical routine in western countries [4] and currently applied volatile anesthetics, e.g. isoflurane, sevoflurane or desflurane, in some cases significantly decelerate the onset of an MH reaction compared to halothane [5,6] and are more likely to lead to abortive MH with eased symptoms. Furthermore, the recommended indications for succinylcholine, another possible MH triggering agent, have been limited by international anesthesia societies [7].

Considering all these facts, the aim of the present study was to investigate, whether MH is still a relevant anesthetic problem in our days.

## Methods

With approval of the local ethics committee (application number: 263/11, ethics committee of the University of Wuerzburg) data of patients who where referred to the MH unit of the Department of Anesthesia and Critical Care of the University of Wuerzburg for diagnostic muscle biopsy and subsequent in vitro contracture testing (IVCT) between 2007 and 2011 were evaluated. Based on available patient documents and medical records the intraoperative events were examined. To confirm the suspicion of an MH crisis, applied triggering agents, clinical symptoms e.g. cardiac arrhythmia, increase of end-tidal carbon dioxide ≥ 45 mmHg, rises of patients’ body temperature ≥ 38.5°C and possible use of dantrolene were analyzed. Besides that, the medical records were reviewed for severe postoperative complications, e.g. neurological deficits, disseminated intravascular coagulation (DIC), acute renal failure or signs of rhabdomyolysis according to maximum creatine kinase (CK) levels. If blood gas analysis was implemented, pH ≤ 7.2, base excess ≤ -5 mmol/l and PaCO_2_ ≥ 50 mmHg defined a severe metabolic response. Only patients with a suspected MH episode during general anesthesia due to the estimation of the responsible anesthesiologist, completed IVCT and genetic analysis of the ryanodine receptor gene were included in the investigation.

In referred patients a diagnostic IVCT with increasing caffeine and halothane concentrations in separated tissue baths was performed according to the guidelines of the European MH Group [8]. A contracture ≥ 2 mN at caffeine 2 mM and halothane 0.44 mM lead to the diagnosis MH susceptible (MHS). If significant contractures occurred after one of the drugs only, patients were classified as MH equivocal (MHE, MHE for halothane (MHEh) or caffeine MHEc). If no significant contracture was observed the patients were rated MH non-susceptible (MHN).

In addition, for each patient, the clinical grading scale (CGS) by Larach and colleagues, which includes metabolic and muscular parameters as well as changes in cardiac rhythm and body temperature, was applied retrospectively. According to the grading scale 3 to 15 points were calculated for each parameter and added to receive a score. This score allowed allocation to individual MH-ranks (0 = MH almost never, 3-9 = MH unlikely, 10-19 = MH somewhat less than likely, 20-34 = MH somewhat greater than likely, 35-49 = MH very likely, > 50 = MH almost certain) [9].

## Results

Between 2007 and 2011 a total of 124 patients underwent a muscle biopsy followed by IVCT at the MH lab of the University of Wuerzburg. Overall 19 of these patients had been referred to the MH unit because of a suspected MH event during general anesthesia on the basis of estimation of the attending anesthesiologists. In the remaining patients MH diagnostics were initiated due to MH susceptibility in the family history, an unexplained rhabdomyolysis or to exclude a myopathic disorder in association with persistently elevated CK levels.

### Diagnostic findings

Muscle biopsy and IVCT detected MH susceptibility in 7 (37%; 7 male) of the 19 patients. In 8 patients (42%; 3 male, 5 female) MH susceptibility could be excluded. Muscle bundles of 4 patients (21%; 1 male, 3 female) developed a pathologic contracture only after exposure to halothane but not after caffeine (MHEh). Interestingly, initial applied CGS rated the probability of an MH crisis as “almost certain” (> 50 points) in 2 MHS patients and “very likely” (35 – 49 points) in 5 MHS and 1 MHEh patients, while in 3 MHEh patients the likelihood was classified as “less than likely” (10 - 19 points). Noteworthy, MH was assumed “greater than likely” (20 – 34 points) in 6 MHN patients and “less than likely” or “almost never” in 1 MHN patient each by CGS. Genetic screening detected mutations in the ryanodine receptor gene (Gly4037Alafs, Glu2174Ala, Val4234Leu) of 3 MHS patients. In 16 (84%) patients the suspected MH event occurred between 2006 and 2010 (6 MHS, 3 MHEh, 7 MHN). The remaining 3 patients had been 10 years old or younger at the time of incident (1992, 1995, 1998) and therefore muscle biopsy in these patients was delayed until the age of 16 years according to our hospital standard operating procedures. Since the MH diagnostic was performed within the study period, these 3 patients were included in the evaluation, even if the applied triggers were halothane or enflurane respectively. Interestingly, 2 MHS individuals with suspected MH in their history had undergone at least one uneventful general anesthesia in the past. The histopathological examinations revealed a myopathic tissue syndrome in combination with cell clumps indicating a possibly neurogenic component in 1 MHEh patient, who had received succinylcholine as sole trigger agent. In the other patients there was no evidence of a muscular pathology (Table 1).

**Table 1:** Diagnostic findings

**No**	**Age/sex**	**IVCT**	**Year of incidence**	**Surgery**	**Prev. Anesth.**	**Genetic status**	**CGS**	**Histopathology**
1	46 ♂	MHS	2010	Liposuction	1	Negative	48	WPF
2	58 ♂	MHS	2010	Spongiosaplasty	2	Gly4037Alafs	38	WPF
3	36 ♂	MHS	2010	Urachal fistula	-	Negative	43	WPF
4	14 ♂	MHS	2009	Lower leg fracture	-	Glu2174Ala	53	WPF
5	18 ♂	MHS	2008	Gunshot injury	-	Val4234Leu	53	WPF
6	45 ♂	MHS	2007	Hemilaminectomy	-	Negative	38	WPF
7	10 ♂	MHS	1995	Appendectomies	-	Negative	40	WPF
8	62 ♀	MHEh	2009	Bursectomy	-	Negative	15	WPF
9	32 ♀	MHEh	2009	Caesarean section	-	Negative	40	Myopathy
10	29 ♀	MHEh	2006	Uterine abrasion	-	Negative	15	WPF
11	3 ♂	MHEh	1992	Tonsillectomy	-	Negative	18	WPF
12	35 ♀	MHN	2011	Uterine abrasion	2	Negative	18	WPF
13	54 ♂	MHN	2010	Aortocoronary bypass	-	Negative	30	WPF
14	46 ♀	MHN	2009	Uterine abrasion	1	Negative	30	WPF
15	34 ♀	MHN	2008	Colon resection	-	Negative	25	WPF
16	57 ♀	MHN	2007	Inguinal hernia	-	Negative	30	WPF
17	21 ♀	MHN	2007	Mandible fracture	-	Negative	0	WPF
18	39 ♂	MHN	2006	Appendectomies	-	Negative	30	WPF
19	3 ♂	MHN	1998	Orchidopexy	-	Negative	30	WPF

*Age* Patients’ age MH-suspected episode occurred, *IVCT* In vitro contracture test, ♂ Male, ♀ Female, *MHS* Malignant hyperthermia susceptible, *MHN* Malignant hyperthermia non-susceptible, *MHEh* Malignant hyperthermia equivocal to halothane, *WPF* Without pathological finding.

### Trigger agents and clinical presentations

Only 21% of the MH suspected patients solely received an inhalation anesthetic (sevoflurane: 1 MHS; isoflurane: 1 MHN; desflurane: 2 MHN), while in 47% of the cases (6 MHS, 1 MHEh, 2 MHN) a combination of succinylcholine with a volatile anesthetic, e.g. halothane (1 MHEh), enflurane (1 MHN) isoflurane (3 MHS, 1 MHN), sevoflurane (2 MHS) or desflurane (1 MHS) was used. In 28% (3 MHEh, 3 MHN) succinylcholine was applied as only MH trigger. Masseter spasm was observed in 63% of the patients (2 MHS, 4 MHEh, 6 MHN), thereof in 28% of the cases (3 MHEh, 3 MHN) after succinylcholine administration. Based on the available patient records, application of volatile anesthetics or succinylcholine was stopped in all of the 19 patients and anesthesia was continued intravenously.

Cardiac arrhythmias were reported in 42% of the 19 cases. Hereof, an unexplained sinus tachycardia with heart rates between 90 to 135 per minutes were documented in 38% of the patients (3 MHS), while in 62% (2 MHS, 1 MHEh, 2 MHN) tachyarrhythmia were observed. In 11% of the patients, who received sevoflurane (1 MHS) or succinylcholine (1 MHEh) solely no arrhythmias were seen. In the remaining suspected cases the cardiac rhythm was not documented in patients’ medical records. An increase of end-tidal carbon dioxide > 45 mmHg during the course of anesthesia was noticed in 42% (5 MHS, 3 MHN). However, body temperature increases ≥ 38.5°C were only reported in 11% of the analyzed cases (1 MHS, 1 MHN). In 47% of the MH suspected cases (7 MHS, 1 MHEh, 1 MHN) an increase of CK levels > 10.000 U/L following MH trigger application was observed. Despite the suspected MH diagnosis, dantrolene was administered only in 37% (5 MHS, 1 MHEh, 1 MHN) for treatment of the observed symptoms (Table 2).

**Table 2:** Applied trigger agents and clinical presentations of malignant hyperthermia suspected events

**No**	**IVCT**	**Trigger agents**	**Masseterspasm**	**Dantrolene application**	**Cardiac arrhythmia**	**Max. end-tidal CO**_**2**_**[mmHg]**	**Max. temperature**	**Max. Creatine kinase [U/L]**
1	MHS	Isoflurane + SCh	Unknown	1 × 200 mg	Sinus tachycardia	48 mmHg	38.8°C	10.514
2	MHS	Isoflurane + SCh	Yes	1 × 240 mg	Tachyarrhythmia	54 mmHg	36.1°C	> 10.000
3	MHS	Sevoflurane + SCh	Yes	No	Sinus tachycardia	Unknown	Unknown	51.557
4	MHS	Sevoflurane + SCh	Unknown	2 × 200 mg	Sinus tachycardia	62 mmHg	Unknown	23.700
5	MHS	Desflurane + SCh	Unknown	1 × 220 mg	Tachyarrhythmia	56 mmHg	37.6°C	≈ 80.000
6	MHS	Sevoflurane	No	1 × 200 mg	No	85 mmHg	37.5°C	38.762
7	MHS	Isoflurane + SCh	Unknown	No	Unknown	Unknown	Unknown	16.412
8	MHEh	SCh	Yes	1 × 200 mg	Unknown	39 mmHg	Unknown	119.150
9	MHEh	SCh	Yes	Unknown	No	36 mmHg	Unknown	162
10	MHEh	SCh	Yes	No	Unknown	Unknown	Unknown	2.234
11	MHEh	Halothane + SCh	Yes	Unknown	Tachyarrhythmia	Unknown	Unknown	Low
12	MHN	SCh	Yes	No	Tachyarrhythmia	Unknown	Unknown	132
13	MHN	Isoflurane	Yes	No	Unknown	54 mmHg	Unknown	4.100
14	MHN	SCh	Yes	No	Unknown	Unknown	Unknown	24.732
15	MHN	Desflurane	No	1 × 180 mg	Tachyarrhythmia	72 mmHg	Unknown	Unknown
16	MHN	SCh	Yes	No	Unknown	Unknown	Unknown	Unknown
17	MHN	Desflurane	No	No	Unknown	Unknown	38,5°C	Unknown
18	MHN	Isoflurane + SCh	Yes	No	Unknown	41 mmHg	Unknown	5.174
19	MHN	Enflurane + SCh	Yes	No	Unknown	75 mmHg	Unknown	4.820

*IVCT* In vitro contracture test, *MHS* Malignant hyperthermia susceptible, *MHN* malignant hyperthermia non-susceptible, *MHEh* Malignant hyperthermia equivocal to halothane, *SCh* Succinylcholine, *CO*_*2*_ Carbon dioxide.

According to the medical records of the referred patients, no persistent or temporary complications e.g. DIC, acute renal failure or neurological deficits were reported during recovery after the suspected MH episode.

### Blood gas analysis

Interestingly, in only 37% of the patients with suspected MH event an arterial blood gas analysis was documented to verify the assumed MH diagnosis. However, a relevant metabolic acidosis with pH ≤ 7.2, base excess ≤ -5 mmol/l and PaCO_2_ ≥ 50 mmHg was observed in 21% (3 MHS, 1 MHN). Besides that, serum potassium levels were remarkable elevated ≥ 5 mmol/l in 16% of the cases (2 MHS, 1 MHN) (Table 3).

**Table 3:** Blood gas analysis of malignant hyperthermia suspected events

**No**	**IVCT**	**pH**	**BE [mmol/l]**	**PaCO**_**2**_**[mmHg]**	**Potassium [mmol/l]**
1	MHS	7.20	-	50	-
3	MHS	7.19	−7	55	3.9
5	MHS*	7.38	1	46	6.3
10	MHS	7.17	−3,8	72	5.0
15	MHN	7.20	−7,9	69	7.3
17	MHN	7.30	-	38	4.1
19	MHN	7.30	-	-	-

*IVCT* In vitro contracture test, *MHS* Malignant hyperthermia susceptible, *MHN* Malignant hyperthermia non-susceptible, *BE* Base excess, *PaCO*_*2*_ Arterial carbon dioxide pressure.

*blood gas analysis after admission at the intensive care unit.

## Discussion

Even though MH is a rare complication of general anesthesia, the presented cases clearly demonstrate that this life threatening muscular hypermetabolism is still a relevant risk requiring immediate and consequent treatment by the responsible anesthesiologist to avoid serious harm to the patient.

After the first description of MH by Denborough numerous cases of fulminant MH as well as in vitro investigations had been published in the following years, identifying halothane and succinylcholine as potential MH triggering agents [10]. While the metabolic deterioration in the course of an MH crisis induced by halothane seems to be a direct consequence of an interaction with the sarcoplasmic ryanodine receptor, the pathophysiological mode of action of succinylcholine has remained unknown. For instance, in vitro succinylcholine increased halothane-induced muscular contractions of MHS patients, but no contracture could be observed after exposition to succinylcholine alone [11]. Even systemic application of succinylcholine could not reproducibly elicit an MH episode in susceptible swine [12,13]. In humans, according to an evaluation of the North American MH Registry and a recently performed European multicentric study, succinylcholine triggered MH in absence of an inhalation anesthetic only in 0.7% or 1% respectively of the investigated cases [14,15]. Since the definitively underlying mode of action of succinylcholine to elicit MH remains unclear so far, the pharmacological characteristics of this agent may enable a possible explanation of it’s role to induce MH. Following intravenous application succinylcholine activates the nicotinergic acetylcholine receptor and provokes a local depolarization of the cell membrane. The transient depolarization of voltage-gated receptors in combination with an influx of extracellular calcium via acetylcholine receptors could lead to a significant increase of intracellular calcium concentrations and after exceeding a certain threshold MH may occurs in affected individuals. In this context, muscular fasciculation and rigidity caused by succinylcholine was considered to be causal for MH. Consequently, a masseter spasm following succinylcholine was postulated to be an early sign of an imminent MH episode. However, specificity of this clinical sign is limited due to the subjective appraisal and the fact, that jaw tightness is a common side effect of succinylcholine, but only in half of the patients associated with MH susceptibility [16]. Similar results were obtained in our investigation. MH susceptibility was confirmed in only 50% of the suspected MH cases, where a succinylcholine-induced masseter spasm was noticed. Interestingly, histological examination of 1 MHEh patient who solely received succinylcholine revealed suspected myopathological finding. Although, neuromuscular disorders are common in MHE patients [17], it remains unclear, if these muscular alterations were responsible for the increased sensitivity to succinylcholine in this patient.

Generally, the likelihood of succinylcholine-induced MH seems to be extremely low, however there is little doubt, that combination with a volatile anesthetic potentiates the onset and the clinical symptoms of an MH event [18]. Remarkably, despite the possibly serious side-effects like MH, hyperkalemia or cardiac arrest, succinylcholine was actually applied to secure the airway in 79% of the referred patients. In part, this approach was reasonable due the higher risk of aspiration in case of trauma or abdominal surgery. However, according to published guidelines the use of the non-depolarizing muscle relaxant rocuronium and if needed followed by application of sugammadex to reverse the neuromuscular blockade might be an adequate alternative to avoid succinylcholine associated adverse effects [7,19].

In contrast to succinylcholine, the impact of all inhalation anesthetics used in daily clinical routine in the development of an MH crisis is beyond dispute. However, dependent on the applied volatile anesthetic the time interval between induction of anesthesia and clinical symptoms of an MH episode seems to vary. For instance, Hopkins and colleagues reported, that in susceptible patients the onset of MH was statistically significant faster after halothane exposure compared to enflurane or sevoflurane [5]. Equally, fulminant MH episodes after isoflurane, sevoflurane or desflurane seem to occur with temporal delay [20,21], while halothane may induce MH within minutes [5]. In MHS animals, similar results were seen after intramuscular injection of halothane or sevoflurane. The induced local hypermetabolic responses measured by local muscular lactate and carbon dioxide pressure increase were more distinct after halothane than after sevoflurane application [22,23]. Furthermore, in vitro the effect on muscular contractures of MHS muscle bundles varies between halothane and modern volatile anesthetics at equivalent concentrations [24]. These different clinical appearances of MH following volatile anesthetic application might be caused due to differences in the calcium releasing potency of these diverse agents. For example, sarcoplasmic calcium release at cellular level was significant smaller after sevoflurane or desflurane exposure compared to equimolar halothane concentrations [25,26]. In the analyzed anesthetic events of the present evaluation, MH episodes were induced by established MH triggers like halothane or isoflurane as well as by modern volatile anesthetics, e.g. sevoflurane or desflurane. Although, in the majority of the cases inhalation anesthetics were combined with succinylcholine and only in one case sevoflurane was applied solely, our findings emphasized the MH trigger potency of newer volatile anesthetics.

Beside masseter spasm cardiac arrhythmias are further early symptoms of imminent MH. Equally to a retrospective analysis from the United States, where the incidence was estimated 40% [14], in the presented investigation the occurrence of unexplained cardiac alterations was 42%. On closer examination the incidence of cardiac symptoms was even higher in the MHS group with either sinus tachycardia or tachyarrythmia as the leading signs.

The low incidence of testified metabolic acidosis might be attributed to the failure to obtain arterial blood gas analysis in the acute phase of the MH reaction or due to dantrolene pretreatment. For example, one patient’s blood gas analysis was performed not until the arrival on the intensive care unit and after treatment with dantrolene, showing an unremarkable blood acid status, while in contrast the intraoperative end-tidal carbon dioxide increased relevant to 56 mmHg in this patient. Overall, in only 37% of the MH suspected cases a blood gas analysis was conducted to verify the suspected diagnosis. This line of action is remarkable, since the presence of an acidosis supports the reasonable suspicion of MH in these cases.

Hyperthermia is a dramatic but often late sign of MH, reflecting the proceeding metabolic breakdown in affected individuals. Hence, temperature monitoring during general anesthesia is recommended if MH triggers are used, since in a couple of cases hyperthermia was the only sign of MH [14]. Fulminant MH episodes may be marked by a rapid increase in body temperature at a rate of 1-2°C every five minutes [27]. Stunningly, only in 11% of the suspected MH cases (1 MHS and 1 MHN) a remarkable hyperthermia with an increase in core temperature ≥ 38.5°C was noticed. The overall low incidence of core temperature rises in the presented study might be attributable to the initiated dantrolene treatment or the possible absence of temperature monitoring.

The pathological changes during MH crisis are based on an uncontrolled increase of myoplasmic calcium, resulting in an ongoing skeletal muscular contracture and loss of cellular integrity leading to hyperkalemia and rhabdomyolysis [28]. Although the surgical trauma itself might cause a significant increase in CK levels, postoperative unexplained excessive hyperCKemia should lead to a diagnostic workup to exclude MH susceptibility as underlying pathology. The reason for the remarkable CK increase up to 24.732 U/L in one of the MHN patients following succinylcholine remains unclear. A not yet diagnosed myopathy could not definitely be excluded, but based on the advanced age of the patient and the inconspicuous histological findings it seems very unlikely.

In contrast to the estimation, that nearly 70% of MH families carry mutations in the ryanodine receptor gene [29], the genetic prevalence of 27% in the analyzed MHS cases was overall low. Noteworthy, even if the Val4234Leu variant of one MHS patient has recently been mentioned in context of a novel exome sequencing method for MH relevant mutations [30], none of the detected genetic variants had been accepted as causative for MH according to the European MH Group database, which includes so far 31 approved mutations of the more than 200 identified ryanodine receptor gene variants [31]. However, it is important to mention, that absence of a causative mutation does not reliably exclude MH susceptibility. To confirm or exclude MH a muscle biopsy followed by an IVCT must be carried out in these patients [32].

After introduction of dantrolene in clinical use a causal treatment of MH has been available since the late 1970’s. The mode of action of this drug is based on inhibition of the sarcoplasmic reticulum calcium release without increasing the reuptake of calcium ions into the sarcoplasmic reticulum [33]. According to current guidelines application of dantrolene is an essential part in the treatment of an MH crisis [34,35]. However, only 37% of the patients in the presented investigation received dantrolene for causal MH therapy. Nevertheless, the importance of consequent dantrolene treatment is absolutely clear [36], even if the hypermetabolic state in some of the presented cases was already terminated by discontinuation of MH trigger substances.

Once surviving fulminate MH episodes several reports documented severe complications, e.g. acute renal failure from rhabdomyolysis, DIC, congestive heart failure or intestinal ischemia due to the uncontrolled metabolic reaction and myocyte death [27]. Fortunately, the review of the medical records of the referred patients, did not detect any serious harms to the patients after an MH episodes, which importantly delayed recovery.

To draw conclusions about the likelihood of MH among the suspected incidents, the “Clinical Grading Scale” (CGS) established by Larach and colleagues assessed clinical and metabolic parameters, e.g. muscle rigidity, rhabdomyolysis, acidosis, increases in body temperature and cardiac arrhythmias [9]. The validity of the CGS may be reduced due to limited availability of complete data sets and hence, often does not satisfactorily correlate with the IVCT results [37]. The false negatives as well as the false positive diagnosis obtained by CGS calculation in our analysis are likely a result of the fragmentary available medical records. Thus, sole evaluation of the CGS seems not to be adequate to prove MH susceptibility.

Finally, anesthesiologists must be aware that uneventful previous general anesthesia does not exclude MH susceptibility [14]. For instance, two of the MHS patients reported a history of exposure anesthesia in the past. The reason why some patients develop MH after first exposition to MH triggering agents, while others do not, still remains unclear and might be explained by an individual cellular compensation mechanism lowering myoplasmic calcium concentrations.

## Conclusions

Analysis of the presented data might be limited by partly incomplete documentation as well as the individual interpretation. Nevertheless, in conclusion MH still is a relevant complication these days and every anesthesiologist must be prepared to recognize the symptoms of MH crisis and to start sufficient treatment. While fulminate courses of MH are easy to diagnose, abortive presentations with solitary or alleviated symptoms are more difficult to detect and pose an enormous challenge to the attending anesthesiologist. The initiation of an adequate and consequent treatment including the application of dantrolene and termination of MH trigger application is essential for patients’ prognosis and survival. Besides that, every patient after a suspected MH event should be referred to a MH center for further counseling.

## Abbreviations

CGS: Clinical grading scale; CK: Creatine kinase; IVCT: In vitro contracture test; MH: Malignant hyperthermia; MHEh: Malignant hyperthermia equivocal to halothane; MHN: Malignant hyperthermia non-susceptible; MHS: Malignant hyperthermia susceptible.

## Competing interests

The authors declare that they have no competing interests.

## Authors’ contributions

FS conceived the study, accompanied the data acquisition, collected and analyzed the data and drafted the manuscript. SJ collected data and helped writing the manuscript. DS collected data. NR participated in the design of the study. All authors read and approved the final manuscript.

## Pre-publication history

The pre-publication history for this paper can be accessed here:

http://www.biomedcentral.com/1471-2253/13/24/prepub
